# Using verbal autopsy to assess the prevalence of HIV infection among deaths in the ART period in rural Uganda: a prospective cohort study, 2006-2008

**DOI:** 10.1186/1478-7954-9-36

**Published:** 2011-08-04

**Authors:** Billy N Mayanja, Kathy Baisley, Norah Nalweyiso, Freddie M Kibengo, Joseph O Mugisha, Lieve Van der Paal, Dermot Maher, Pontiano Kaleebu

**Affiliations:** 1 MRC/UVRI Uganda Research Unit on AIDS. P.O.Box 49, Entebbe, Uganda; 2 MRC Tropical Epidemiology Group, London School of Hygiene and Tropical Medicine, Keppel Street, WC1E 7HT, UK; 3 International Rescue Committee, Tanzania Office, P.O Box 106048, Dar es Salaam, Tanzania; 4 London School of Hygiene and Tropical Medicine, Keppel Street, WC1E 7HT, London

## Abstract

**Background:**

Verbal autopsy is important for detecting causes of death including HIV in areas with inadequate vital registration systems. Before antiretroviral therapy (ART) introduction, a verbal autopsy study in rural Uganda found that half of adult deaths assessed were in HIV-positive individuals. We used verbal autopsy to compare the proportion of HIV-positive adult deaths in the periods before and after ART introduction.

**Methods:**

Between 2006 and 2008, all adult (≥ 13 years) deaths in a prospective population-based cohort study were identified by monthly death registration, and HIV serostatus was determined through annual serosurveys. A clinical officer interviewed a relative of the deceased using a verbal autopsy questionnaire. Two clinicians independently reviewed the questionnaires and classified the deaths as HIV-positive or not. A third clinician was the tie-breaker in case of nonagreement. The performance of the verbal autopsy tool was assessed using HIV serostatus as the gold standard of comparison. We compared the proportions of HIV-positive deaths as assessed by verbal autopsy in the early 1990s and the 2006-2008 periods.

**Results:**

Of 333 deaths among 12,641 adults of known HIV serostatus, 264 (79.3%) were assessed by verbal autopsy, of whom 59 (22.3%) were HIV-seropositive and 68 (25.8%) were classified as HIV-positive by verbal autopsy. Verbal autopsy had a specificity of 90.2% and positive predictive value of 70.6% for identifying deaths among HIV-infected individuals, with substantial interobserver agreement (80.3%; kappa statistic = 0.69). The HIV-attributable mortality fraction estimated by verbal autopsy decreased from 47.0% (pre-ART period) to 25.8% (ART period), p < 0.001.

**Conclusions:**

In resource-limited settings, verbal autopsy can provide a good estimate of the prevalence of HIV infection among adult deaths. In this rural population, the proportion of deaths identified by verbal autopsy as HIV-positive declined between the early 1990s and the 2006-2008 period. Verbal autopsy findings can inform policy on HIV health care needs.

## Background

Most resource-limited settings have inadequate or no vital registration systems, although many deaths in these areas occur outside health care facilities and vital registration data are essential for public health planning [1,2]. Since 2004, many countries including Uganda have scaled up access to antiretroviral therapy (ART). Knowing the impact of ART introduction on the proportion of deaths that are associated with HIV is important for policymakers [3,4].

The World Health Organization (WHO) has stimulated interest in the use of verbal autopsy as a tool to obtain information on causes of death in areas with inadequate vital registration systems, for public health planning and resource allocation [5]. In a WHO workshop, priority research questions were identified for the use of verbal autopsy tools in longitudinal population-based HIV studies: the accuracy of verbal autopsy for HIV/AIDS identification, especially in terms of detecting changes in causes of death over time; the best verbal autopsy questions to identify HIV infection in deceased persons; and verbal autopsy questions to be used for ART-related deaths [6].

From 1990 to 1993, before ART was available in rural Uganda, the verbal autopsy tool was validated using the HIV test results obtained from consecutive annual HIV serosurveys in the same population. Half of the deaths among adults aged 13 years and above were among HIV-positive people and the specificity and positive predictive value of the verbal autopsy tool for identifying HIV-positive deaths were both 92%. The study methods and verbal autopsy questionnaire used during this study have been described in detail before [7].

We designed a study to evaluate the usefulness of verbal autopsy in identifying deaths among adults with HIV infection after the introduction of ART in 2004 in the same population. We also compared the proportions of deaths identified as HIV-positive before and after ART introduction, by comparing our results to those from the 1990-1993 verbal autopsy study.

## Methods

### Study setting and participants

In 1989, a general population cohort (GPC) was established in rural southwest Uganda to describe the population dynamics of HIV infection. The GPC originally consisted of around 4,500 adults (aged 13 years and above) in 15 neighboring villages [8]. In 1999, 10 more villages were added to the survey area, bringing the total number of adults to around 14,530 in 2006-2008 [9]. Annual house-to-house census and HIV serosurveys are conducted. The average annual survey participation is 60% to 65%, although 84% have ever participated in the HIV serosurvey. In 1990, a clinical cohort nested within the GPC was established to study the natural history of HIV infection. The study setting has a stable homogeneous population, in which adult HIV prevalence initially declined from 8.5% in 1990-1991 to 6.2% in 1999-2000 but then rose to 7.7% in 2004-2005 [10,11]. Since January 2004, ART has been provided for all eligible participants at the study clinic according to the Uganda National ART guidelines [12,13]. At the time of the study, the population of Uganda was 29.6 million people with a national average HIV prevalence of 6.4% [14].

#### Notification of deaths and condolence visit

The GPC study purposely instituted 26 community-based recorders, who every month collect and report information on all deaths and births that occur in their villages in the study area, and this information is updated by the annual census conducted in the same cohort. From January 2006 until December 2008, all deaths that occurred in adults in the study area were assessed by verbal autopsy. A home visitor paid a condolence visit to relatives of the eligible deceased soon after the death (usually within one month) and informed them about the study, giving them opportunity to ask questions. A copy of the study information sheet was given to the relatives most closely associated with the deceased. A verbal autopsy interview appointment was made (approximately two months after death occurred) with consenting relatives.

### The verbal autopsy visit

The interviewer (a clinical officer) and a home visitor visited the relative of the deceased on the agreed date and, after obtaining written informed consent, administered a verbal autopsy questionnaire. The verbal autopsy questionnaire was the same as that validated in 1990-1993 in the same study area but with additional questions on ART use (Appendix). The questionnaire was comparable to the WHO SAVVY (sample vital registration with verbal autopsy) International verbal autopsy questionnaire 3 for death of a person aged 15 years and above. However, it missed some of the questions in Section 5: History of previously known medical conditions (especially high blood pressure, diabetes, asthma, and cancer), Section 7: Symptoms and signs associated with illnesses of women, Section 8: Symptoms and signs associated with pregnancy, and Section 10: Treatment and health service use for the final illness.

During the verbal autopsy interview, the respondent was asked to give a narration of the events preceding the death and then specific questions were asked on the symptoms prior to death and whether the deceased was on ART. At the end of the verbal autopsy interview, the opinion of the respondent on the deceased's cause of death was sought by asking whether the respondent thought that he/she knew the cause of death. If the response was 'yes', the respondent was asked how or from whom he/she knew (for example, own opinion, from another household member, from doctor/nurse treating the deceased). The interviewer also recorded what she thought was the cause of death. Each completed questionnaire was checked for completeness and consistency by a physician and any queries were discussed and clarified with the interviewer.

### Independent allocation of cause of death

The verbal autopsy questionnaires were independently reviewed by two experienced physicians (both clinical epidemiologists) from outside the study area to ascertain the likely cause(s) of death and whether the deceased was HIV-positive. There were no specified criteria given to the physicians upon which to make the assessment as to whether the deceased was HIV-positive or not; physicians were allowed the freedom to use their clinical knowledge of HIV disease presentation. The assessing physician had access to the respondent's opinion on the cause of death. In case of nonagreement between the two clinicians on whether the deceased was HIV-positive or not, the opinion of a third, more senior physician (an internist) was sought. The physicians recorded their findings on specifically designed verbal autopsy forms.

### Sample size

We aimed to assess by verbal autopsy all deaths that occurred in the study population between 2006 and 2008. No formal sample-size calculations were done.

### Statistical methods

Data were double-entered into an MS Access database (Microsoft Corp., USA) and analyzed using Stata version 10 (Stata Corporation, College Station, Texas, USA). The analysis of verbal autopsy data was restricted to deaths in individuals who had participated in at least one annual census round and so had a valid unique census identification number. This identification number was used to obtain the individual's HIV serostatus by linkage with the annual HIV serosurvey results in the population cohort database. We assessed the performance of the verbal autopsy tool in identifying HIV infection among deaths in adults aged 13 years and older. Since we did not have data on the actual cause of death, we defined as "HIV-associated" any death occurring in a person who was HIV-seropositive.

We calculated specificity and positive predictive values of the verbal autopsy tool, using HIV serostatus as the gold standard. True positive was defined as HIV-positive status, and true negative was defined as HIV-negative status at the last available serosurvey. A verbal autopsy test positive was defined as an HIV-associated diagnosis by both assessors, or two out of three assessors in the case of nonagreement between the first two. A verbal autopsy test negative was defined as an HIV-negative diagnosis, or unknown HIV association, by two out of three assessors. We assessed interobserver agreement between the first two assessors using a kappa statistic. We calculated HIV-attributable mortality fraction as the proportion of deaths of known HIV serostatus that were classified as "HIV-positive" by verbal autopsy.

### Ethical aspects

The study was approved by the Science and Ethics committee of the Uganda Virus

Research Institute and the Uganda National Council for Science and Technology. Informants gave written informed consent prior to participating in the study. A condolence fee of 5,000 Uganda shillings (US$ 2.20) was given to each informant.

## Results

Between January 2006 and December 2008, there were 387 deaths in the study area among 14,530 adult participants aged 13 and older in the GPC census and 333 deaths among 12,641 adults with known HIV serostatus (mortality rate = 10.8 per 1000 person-years, 95% confidence interval (CI): 9.8, 12.1). Among the 333 deaths of known HIV status, verbal autopsy was done for 264 (79.3%). Of the 69 deaths of known HIV serostatus where verbal autopsy was not done, in 29 (42%) there was no relative to interview, in 15 (22%) the relative refused to be interviewed, and in 25 (36%) no reason was given.

Of the 333 deaths of known HIV status, a similar proportion of HIV-positive (78.7%) and HIV-negative (80.1%) deaths were assessed by verbal autopsy (Table 1). Among the 264 deaths assessed by verbal autopsy, 59 (22.3%) were HIV-positive and had a mean (SD) age of 41.0 (14.4) years while 205 (77.7%) were HIV-negative with a mean (SD) age of 60.1 (22.8) years. Among all HIV-seropositive deaths in the GPC, there was no evidence of a difference in mean age between deaths assessed by verbal autopsy and those that were not (p = 0.78). However, among the HIV-seronegative deaths in the GPC, there was some evidence that the mean age of deaths assessed by verbal autopsy was greater than that of deaths that were not assessed (61.4 versus 54.9 years, respectively, p = 0.06).

**Table 1:** Total deaths of known HIV serostatus and available verbal autopsy (VA) deaths, by age and HIV status.

Age group (years)	VA assessed/total (%)		
	
	HIV-positive	HIV-negative	Deaths known HIV status
13-24	4/7 (57.1%)	19/29 (65.5%)	23/36 (63.9%)
25-34	20/24 (83.3%)	19/22 (86.4%)	39/46 (84.8%)
35-44	12/16 (75.0%)	13/20 (65.0%)	25/36 (69.4%)
45-54	12/13 (92.3%)	12/16 (75.0%)	24/29 (82.8%)
55-64	7/9 (77.8%)	28/36 (77.8%)	35/45 (77.8%)
65+	4/6 (66.7%)	114/135 (84.4%)	118/141 (83.7%)

**Total**	**59/75 (78.7%)**	**205/258 (80.1%)**	**264/333 (79.3%)**

**Mean (SD) age (years)**			
VA assessed	41.2 (14.0)	61.4 (22.0)	56.9 (22.1)
Not assessed	40.1 (16.2)	54.9 (25.2)	51.5 (24.2)
**All deaths**	**41.0 (14.4)**	**60.1 (22.8)**	**55.8 (22.6)**

Overall, 68 (25.8%) deaths were classified as HIV-positive by verbal autopsy. The proportion of deaths that were classified as HIV-positive decreased with increasing age: from 50.6% in those aged 13 to 44 years to 5.1% among those aged 65 years and older (Table 2). Similarly, the proportion of true HIV-seropositive deaths decreased with age, from 41.4% of those aged 13 to 44, to 3.4% of those aged 65 years and older. The verbal autopsy had an overall specificity of 90.2% and positive predictive value of 70.6% for diagnosis of HIV-positive death. In participants aged 13 to 44 years, the corresponding values were 76.5% and 72.7%, respectively, while among those aged 65 years and over, the values were 96.5% and 33.3% respectively (Table 3).

**Table 2:** Verbal autopsy classification by age and HIV serostatus.

	HIV status by verbal autopsy classification	Total (%)	True HIV serostatus	
			
			HIV-positive	HIV-negative
13-44 years	Positive	44 (50.6%)	32	12
	Negative	32 (36.8%)	2	30
	Don't know	11 (12.6%)	2	9
		**87 (100%)**	**36 (41.4%)**	**51 (58.6%)**
45-64 years	Positive	18 (30.5%)	14	4
	Negative	35 (59.3%)	4	31
	Don't know	6 (10.2%)	1	5
		**59 (100%)**	**19 (32.2%)**	**40 (67.8%)**
65+ years	Positive	6 (5.1%)	2	4
	Negative	103 (87.3%)	1	102
	Don't know	9 (7.6%)	1	8
		**118 (100%)**	**4 (3.4%)**	**114 (96.6%)**
**All ages**	Positive	68 (25.8%)	48	20
	Negative	170 (64.4%)	7	163
	Don't know	26 (9.8%)	4	22

**Total deaths**		**264 (100%)**	**59 (22.3%)**	**205 (77.7%)**

**Table 3:** Performance of the verbal autopsy tool using HIV serostatus as gold standard, by age group

	Age group (years)			
	
	13-44	45-64	65+	All ages
Specificity (95% CI)	76.5% (62.5-87.2%)	90.0% (76.3-97.2%)	96.5% (91.3-99.0%)	90.2% (85.3-93.9%)
Positive predictive value (95% CI)	72.7% (57.2-85.0%)	77.8% (52.4-93.6%)	33.3% (4.3-77.7%)	70.6% (58.3-81.0%)
HIV-attributable mortality fraction (95% CI)				
this study	50.6% (39.6-61.5%)	30.5% (19.2-43.9%)	5.1% (1.9-10.7%)	25.8% (20.6-31.5%)
cohort (based on ALPHA data^2^)	40.3%	24.4%	1.6%	17.0%

^1^Test (verbal autopsy) positive defined as positive by both assessors, or by 2 out of 3 assessors in case of nonagreement; test negative defined as negative or unknown by both assessors, or by 2 out of 3 assessors in case of nonagreement.

^2^Unpublished Analysing Longitudinal Population-based HIV cohorts in Africa (ALPHA) data

The assessors' agreement as to whether the death was HIV-positive was found in 212 (80.3%) deaths (kappa statistic 0.69). The 52 discordant assessments were evenly distributed between deaths of participants who were HIV-positive (20.3%) and negative (19.5%) (p = 0.89). The HIV-attributable mortality fraction estimated by verbal autopsy decreased from 47.0% (pre-ART period) to 25.8% (ART period) (p < 0.001; Table 4).

**Table 4:** Comparison of verbal autopsy-assessed HIV-associated deaths in pre-ART and ART periods.

Characteristics	Verbal autopsy study period	
Study period	Pre-ART (1990-93)	ART (2006-08)

HIV prevalence	8.0%	7.5%

Deaths of known HIV serostatus	293	333

Deaths where verbal autopsy was done	155 (53%)	264 (79%)

HIV-positive deaths with verbal autopsy	78 (50%)	59 (22%)

Specificity	92%	90%

Positive predictive value	92%	71%

Assessors' agreement on HIV-associated deaths	91%	80%

Kappa statistic	n/a	0.69

HIV-attributable mortality fraction-VA study	47%	25.8%

HIV-attributable mortality fraction-cohort data	50%	17%

## Discussion

In this study population, which has no official vital registration system, we found that verbal autopsy had an overall specificity of 90% and positive predictive value of 71% in identifying HIV infection among adult deaths. Since the introduction of ART in 2004, the prevalence of HIV infection among adult deaths, as estimated by verbal autopsy, has reduced, with the HIV-attributable mortality fraction falling from 47% in the early 1990s to 25.8% in 2006 to 2008.

We found that verbal autopsy performed well in identifying HIV-positive deaths in the age groups 13 to 44 years and 45 to 64 years. This finding is in agreement with findings from a study conducted in Tanzania and Zimbabwe, which showed that, in generalized HIV epidemics, verbal autopsy can be used to reliably measure AIDS mortality, especially in the 15 to 44 year age group [15]. The positive predictive value of verbal autopsy is lower among people aged 65 and older than in the younger age groups because of the lower prevalence of HIV-positive deaths. In our study, only 3% of the deaths in those 65 years and older were HIV-positive. In contrast, a study in Kenya reported that AIDS was the principle cause of death among older people up until the age of 70 years [16]. In populations such as ours with a lower prevalence of HIV-positive deaths among people over 65, improved verbal autopsy tools are needed to reliably measure HIV-positive deaths in this age group.

In 2006 to 2008, during the time of this verbal autopsy study, the estimated national ART coverage in Uganda was 33% [17]. As expected, when compared to the earlier verbal autopsy study in the period before ART was introduced in this community [7], we found a decline in the proportion of deaths that were HIV-positive. Our estimated HIV-attributable mortality fraction from the verbal autopsy data is somewhat higher than that estimated from the main GPC cohort based on HIV serostatus (25.8% versus 17.0%). The observed decline in HIV-attributable mortality fraction since the early 1990s reflects improved clinical care and cotrimoxazole prophylaxis before ART was available, as well as the introduction of ART in 2004. The impact of ART in reducing HIV-associated mortality has been documented before [18-20]. Access to ART has improved the survival of HIV-infected individuals, making HIV infection a chronic manageable disease and allowing these individuals to live socially- and economically-active lives [21]. Although worldwide the number of people living with HIV increased from 29 million in 2001 to 33.4 million in 2008, HIV-associated deaths increased only from 1.9 million to 2 million over the same period [18]. We assessed a higher proportion of all adult deaths by verbal autopsy than was done in the 1990-1993 study. However, in both studies, there was no difference by HIV status in the proportion of deaths assessed. Therefore, our observed decrease in the estimated HIV-attributable fraction is unlikely to be a result of HIV-positive selection bias in the verbal autopsy assessed deaths.

In this study we used two physicians to review and independently ascertain whether the deceased was HIV-positive or not, and a tie-breaker third clinician in case of nonagreement. There has been debate as to which is the best method to use to ascertain the cause of death from verbal autopsy questionnaires in resource-limited settings. Though physician review of verbal autopsy questionnaires is the most commonly used method, issues have been raised about the possible inter- and intra-observer variability, the demands on physicians' time, and the remuneration costs of these physicians [22,23]. Other techniques like the probabilistic (InterVA) model [24,25], symptom pattern [26], machine learning, and tariff method have been developed and validated, but a universally accepted method is yet to be decided.

The assessing clinicians' ascertainment of whether the deceased was HIV-positive could have been biased by the opinion of the deceased's relative. Without access to this information, the performance of the verbal autopsy tool may have been lower, and our results may not be generalizable to settings where the respondent's opinion was not available. However, the majority of deaths in our cohort occurred at home or outside health care facilities without medical attention, and so the responses of caregivers and relatives may reflect general awareness of HIV and recognition of its symptoms in the community. Thus our results may be generalizable to other rural settings with similar HIV prevalence, in which verbal autopsy information is obtained from family members.

This study had several strengths. The annual HIV survey allowed the HIV serostatus of the majority of deaths to be known. The monthly reports of deaths in the area by the community-based recorders allowed us to have a fairly complete death registration. We ensured consistency in the interviewing process by using the same interviewer for all the relatives of the deceased. The use of the same questionnaire as in the previous study enabled comparison of the findings of the two studies. There was substantial interobserver agreement between the two assessing clinicians and verbal autopsy had good specificity and positive predictive value for the identification of HIV-positive deaths. The two-month interval between death and the verbal autopsy interview likely minimized any difficulty participants had in recalling the circumstances of their loved ones' deaths and minimized the stress caused by the verbal autopsy interview, resulting in a high participation rate.

Our study limitations included the fact that some of the censused GPC participants had not participated in the annual HIV serosurvey and therefore had no HIV test results and were excluded from analysis. Furthermore, some individuals may not have participated in a recent serosurvey and thus may have seroconverted since their last negative HIV test. However, the HIV incidence in this population is relatively low (4.3, 95% CI: 3.6, 5.1 per 1,000 person-years; unpublished data), so any misclassification bias is likely to be small. We also could not carry out verbal autopsy for all the deceased participants as some relatives refused to be interviewed and, in other cases, there were no relatives to interview. Using HIV status (instead of the actual cause of death) as the comparison gold standard for HIV-associated deaths may not be ideal, as some HIV-infected participants might have died of other non-HIV-related causes.

## Conclusions

The good specificity and positive predictive value of verbal autopsy in this study in identifying deaths in HIV-positive persons in a resource-limited setting with limited official vital registration systems adds to the evidence of the value of verbal autopsy in determining HIV-positive deaths. Policymakers should consider verbal autopsy as a source of information about deaths occurring among HIV-positive individuals and HIV-associated deaths that can be added in routine health information systems.

## List of abbreviations

ART: antiretroviral therapy; GPC: general population cohort

## Competing interests

The authors declare that they have no competing interests.

## Authors' contributions

BNM was involved in data acquisition and data interpretation and wrote the first draft of the manuscript; KB was involved in data analysis and interpretation; NN, FMK, and JOM were involved in data acquisition and interpretation; LVP was involved in the study conception and design, data acquisition, and interpretation; DM was involved in data interpretation; PK was involved in the study conception and design, data acquisition, and interpretation and was the overall custodian of the study. All authors were involved in the preparation of the manuscript and have read and given approval of the final version.

## Appendix

Items included in ART period (2006 - 2008) verbal autopsy study questionnaire.

General

•Where death occurred

• Any spouse of the deceased died in the last 5 years, if so, believed cause of death

• Respondent's detailed account of illness

Circumstances of death

• Duration of terminal illness

• Was death sudden and unexpected

• Type of medical treatment during the last 3 months before death

• Whether deceased was on ART, and if yes, when ART was started

• Ever treated for tuberculosis

• Health unit(s) attended

• Records available in home (extract findings)

Signs, symptoms and their severity during the last illness

• Fever

• Diarrhea

• Dehydration

• Vomiting/associated abdominal pain

• Breathing (chest pain/chest in-drawing/difficult/rapid/wheezing)

• Cough (severe/productive/blood in sputum/followed by vomiting)

Neurological

• Neck stiffness

• Unconscious

• Fits (jerking of whole body/individual limbs/frequency per day)

• History of epileptic illness in earlier years

• Paralysis of limbs

• Rigid body stiffness, unable to open mouth

Others

• Skin rash and itching

• Red and sore eyes

• Loss of weight

• Abscesses/body sores

• Body swelling (edema/which parts)

• Constipation (associated abdominal pain)

• Hair changes (light colored or thin)

• Yellowing of eyes/passing brown urine

• Herpes zoster (at any time in life)

Respondent's opinion of cause of death

• Specify

• How or from whom did you know

Interviewer's diagnosis

• Illness 1

• Illness 2

Final classification (Clinician)

• Death HIV-related

• Death ART-related

• Leading cause of death

• Subsidiary causes
